# Digital Ischemia after Ultrasound-Guided Alcohol Injection for Morton’s Syndrome: Case Report and Review of the Literature

**DOI:** 10.3390/jcm11216263

**Published:** 2022-10-24

**Authors:** Carlo Biz, Barbara Bonvicini, Giovanni Sciarretta, Mattia Pendin, Giovanni Cecchetto, Pietro Ruggieri

**Affiliations:** 1 Orthopedics and Orthopedic Oncology, Department of Surgery, Oncology and Gastroenterology DiSCOG, University of Padova, Via Giustiniani 3, 35128 Padova, Italy; 2 Department of Cardiac, Thoracic, Vascular Sciences and Public Health, Legal Medicine, University of Padua, Via Gabriele Falloppio 50, 35128 Padova, Italy

**Keywords:** Morton’s neuroma, Morton’s syndrome, alcoholization, alcohol injection, ischemia, digital amputation

## Abstract

The therapeutic algorithm for symptomatic Morton’s syndrome is not standardized as several managements have been proposed. Ultrasound-guided alcohol injection (USGAI) is one of the non-operative procedures described. This report presents the case of digital ischemia that occurred after alcoholization for the treatment of Morton’s syndrome. This complication is described for the first time in the scientific literature, and it should not only be added to the list of sequalae of USGAI, but more importantly, it should also be explained to the patient when this alternative treatment to traditional surgery is proposed.

## 1. Introduction

Morton’s syndrome (MS) is an entrapment degenerative neuropathy that most commonly involves the third interdigital nerve [1] and is one of the most frequent causes of neuropathic chronic forefoot pain. This condition was first anatomically described by Civinini in 1835 and subsequently clinically described by Thomas Morton in 1876 [2,3].

Its prevalence is estimated to be 88 women every 100,000 and 50 men every 100,000 [4], most commonly between the fourth and sixth decade of life. It is characterized by persistent painful swelling of the common digital plantar nerve (CDPN), known as a “neuroma” [5]. However, this is a misnomer, as the lesion consists of peri-neural fibrosis with no neoplastic tissue.

Its etiopathogenesis is still not clear. Different theories have been proposed: entrapment, chronic trauma, intermetatarsal bursitis, and ischemia [6]. The most common hypothesis considers MS as a canalicular syndrome due to the conformation and functional complexity of the distal intermetatarsal region, which is a stiff osteofibrous channel. Stecco and colleagues [7] hypothesized that alterations in foot support and altered biomechanics act on the interosseous muscles, increasing the stiffness of the dorsal fascia, particularly at the points where these muscles are inserted. Chronic rigidity of this fascia increases the stiffness of the inter-metatarsal space, leading to entrapment of the CDPN. Further, the same authors have reported more recently that the deep fasciae are very well innervated [8,9], and their alteration can be a further source of neuropathic chronic pain. Hence, stiffness of fascia in the foot could also irritate the free nerve endings inside the fascial tissue, causing an additional source of nociceptive pain [10,11].

Histologically, Morton’s neuroma (MN) is characterized by hyaline degeneration of the endoneurium, endoneurial edema, and endo-perineural connective tissue deposition due to the widespread presence of an amorphous, eosinophilic substance enveloping a few small cells [7].

Clinically, this syndrome presents with a severe burning sensation, paresthesia, sharp metatarsalgia, numbness, and stabbing pain in the inter-metatarsal plantar region, which can also spread to adjacent toes, the dorsum of the foot, and the hindfoot. These symptoms worsen by walking and wearing tight-fitting shoes [12,13]. Physical examination reveals pain on palpation of the affected region, a positive Mulder’s maneuver with a palpable click, and the squeeze test [5], despite the lack of any specific test for determining the presence of a Morton’s neuroma.

Diagnosis is mainly based on clinical presentation and physical examination. Ultrasound and magnetic resonance imaging (MRI) [14,15], even if they demonstrate high false negative values and appear to be relevant only when the size of the neuroma exceeds 5 mm in transverse diameter [16], are both useful for the morphological study of the lesion to exclude other causes of metatarsalgia and to confirm the diagnosis.

The therapeutic protocol for symptomatic MS is not standardized and includes several treatments [17]: conservative methods, minimally invasive techniques, and surgical procedures. Among non-surgical interventions, the following have been described: corticosteroid injection, extracorporeal shockwave therapy (ESWT), radiofrequency ablation (RFA), cryoablation, capsaicin injection, botulinum toxin, wider footwear and metatarsal padding, fascial manipulation, yttrium aluminum garnet (YAG) laser therapy, and alcohol injection [18,19]. First-line management is nonoperative [4]. The most reliable treatments include lifestyle modifications, such as avoidance of tight-fitting shoes, orthotics, local infiltrations of corticosteroids [18,20,21,22], as well as mobilization and manipulation techniques [23,24,25,26]. Surgical intervention, recommended after failure of conservative therapies, includes neurectomy, i.e., excision of the affected CDPN segment, and neurolysis. The latter consists of a section of the dorsal fascia of the foot and the deep transverse ligament. Both techniques seem to guarantee the best outcomes for patients especially in the long term, still with a considerable probability of complications [5,14,27,28,29]. Additionally, Lee et al. [30] found that with long-term follow-up of patient outcomes (minimum of 10 years) after neuroma excision, patients demonstrate progressive worsening as compared with mid-term and short-term results.

The alcohol injection technique is based on chemical neurolysis by means of dehydration, necrosis, and precipitation of protoplasm [31]. In 1999, Dockery first proposed percutaneous alcohol sclerosing injection as a valid alternative treatment in 100 adult patients with intermetatarsal neuroma. The enrolled subjects received from 3 to 7 injections of 0.5 mL of a 4% alcohol sclerosing solution by using a 27-gauge needle in the intermetatarsal space involved at intervals of 5 to 10 days. Although the procedure success rate was reported to be around 82%, a temporary sensation of increased symptoms after the first injection was described in the first 48 h [32]. Since then, numerous studies have been performed, and all have reported satisfying outcomes [33]. Alcoholization is usually performed under ultrasound (US) guidance in order to improve intralesional injection and produce better long-term outcomes [34]. This is not a standardized technique: indications often are not clear, and the alcohol concentration used (between 4% and 50%) varies according to the clinician’s experiences, as does the volume infiltrated (from 0.4 mL to 1.0 mL) [32,35]. Although the therapeutic alcohol injections are considered a simple, effective, and safe procedure, various adverse events have been reported, mostly transient and minor: phlogistic reactions, transitory plantar pain due to a subtle leakage of fluid [33], forefoot bone marrow edema [36], severe burning post-injection, worsening of symptoms, hypo/anesthesia of the innervated area [37], skin necrosis [38], and allergic reaction [39].

In this article, we report a rare case of digital ischemia at the level of the fourth toe after ultrasound-guided alcohol injection (USGAI) for MS treatment and subsequent digital amputation. To our knowledge, this is the first published report on this specific complication following this procedure for plantar digital compressive neuropathy syndrome.

## 2. Case Presentation

**Figure 1:**
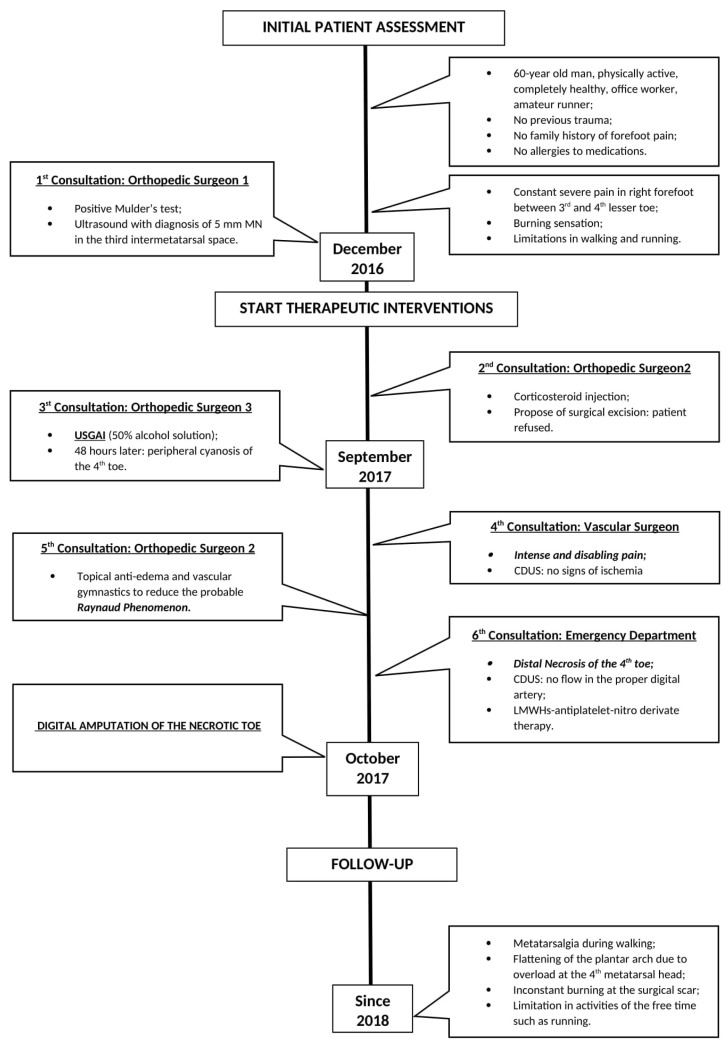
The Flow Chart of the Case Report.

The current case report was described in accordance with the Consensus-based Clinical Case Reporting Guideline as proposed in 2014 [40] and following the Flow Diagram for Case Reports as update in 2019 [41] (Figure 1).

The patient, a 60-year-old man, an office worker by profession and amateur runner during his free time, sought medical attention with complaints of constant and severe pain in his right forefoot, associated with a burning sensation on the plantar surface for 2 years. He described these symptoms located plantarly between the third and the fourth lesser toe, radiating to the phalanges and exacerbated by walking. Sometimes the pain became constant and so intense, also accompanied by swelling and cramping in the forefoot, as to prevent him from walking and running, and forcing him to remove his shoe to reduce the symptoms.

In December 2016, during a first orthopedic examination at the local hospital (first consultation), a positive Mulder’s maneuver was observed, and then a right foot ultrasound was performed, identifying a 5 mm hypoechoic nodule in the third intermetatarsal space, compatible with MN. At the time of the presentation of symptoms, the patient was completely healthy, physically active, and without a family history of forefoot pain. He had no allergies to medications. He did not drink or smoke and denied any drug use. He did not report a specific history of previous trauma. During a following evaluation elsewhere (second consultation), a corticosteroid injection was performed to relieve symptoms temporarily as the diagnostic suspicion had been confirmed, and surgical excision was proposed, which the patient refused.

**Figure 2:**
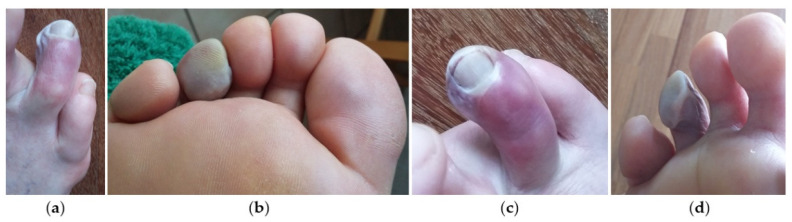
Plantar and dorsal view of skin conditions with progressive ischemia of the fourth toe after USGAI. Clinical images at 10 days (**a**,**b**) and 20 days (**c**,**d**) from the procedure.

After a few months, he decided to be evaluated in a different clinic in another city (third consultation), where the consulted orthopedic surgeon suggested a cycle of three USGAIs (50% alcohol solution and 50% anesthetic, lidocaine 2% solution) on the third intermetatarsal space, which the patient accepted. During this procedure the patient was in supine position with the knee flexed 45 degrees and the forefoot resting on a roll made of folded sheets. The operator, the same orthopedic surgeon, using a 25-gauge needle, injected the solution inside the enlarged neuroma through a dorsal approach and after localization of the neuroma under US guidance by an experienced radiologist. However, the exact volume of the injected solution, usually varying from 0.6 to 1.0 mL depending on the size of the neuroma [35,37], was not reported in the patient’s clinical notes at the time of USGAI. The patient was discharged immediately after the percutaneous procedure and rest was recommended for the first few days along with local ice packs for the first 2 days. Less than 48 h after the injection, as a consequence of the appearance of peripheral cyanosis on the right fourth toe and the persistence of acute pain in the injection site, he decided to inform the Orthopedic Surgeon who had performed the procedure (Figure 2).

The patient was reassured that it was only a transient complication caused by the injection and the bluish color of the fourth toe would soon be gone. During the following days, the pain became so intense and disabling that he could not sleep lying down. Hence, he opted for another consultation by a vascular surgeon of the local city hospital (fourth consultation) who performed a color duplex ultrasound (CDUS), which showed no signs of ischemia: specifically, the dorsal pedis artery and the common digital artery of the fourth toe were patent. The patient then decided to be re-examined by the Orthopedic Surgeon (fifth consultation) who first suggested surgical excision (during the second consultation). The surgeon recommended the use of topical anti-edema and vascular gymnastics to reduce the probable Raynaud phenomenon and proposed surgical treatment again to be performed when skin conditions were improved.

**Figure 3:**
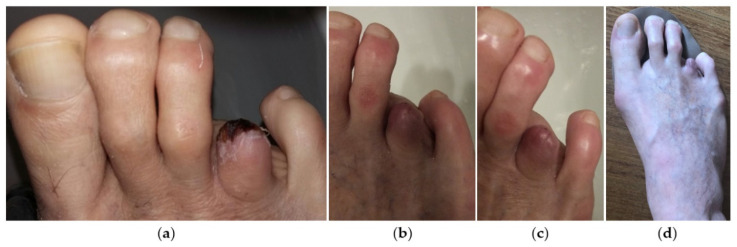
Clinical images of the right forefoot after digital amputation during post-operative period (**a**–**c**) and at the time of medical legal assessment (**d**).

Because of worsening of symptoms a few days later, the patient was admitted to the emergency department (ED) of his local hospital (sixth consultation). First, he was evaluated by an orthopedic consultant, who diagnosed apical necrosis of the toe and then by a vascular surgeon, who performed CDUS that demonstrated no flow in the proper digital artery of the fourth intermetatarsal space. The patient was then discharged; combined anticoagulant (LMWHs)-antiplatelet-nitro derivative therapy was recommended, and a following outpatient vascular reevaluation was programmed. Finally, ten days later (about 40 days from USGAI), the vascular surgeon suggested a digital amputation of the necrotic fourth toe, which the patient accepted (Figure 3).

**Figure 4:**
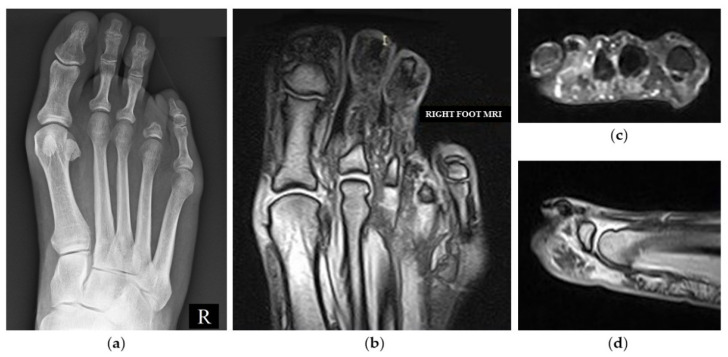
Weightbearing X-ray AP image of the right foot showing the fourth toe amputation at the level of the base of the P1 after surgery (**a**). MRI of the right forefoot performed at 6 months from the amputation, showing flattening of the plantar arch, signs of overload of the fourth metatarsal head and persistence of suprafascial plantar edema (**b**) coronal; (**c**) axial; (**d**) sagittal view.

From that day, the patient takes lysine acetylsalicylate 75 mg daily for preventive purposes, as suggested by the vascular surgeon. At a medical legal assessment held one year after the event as a consequence of the digital amputation, the biomechanics of the foot was radically changed, leading to metatarsalgia of significant intensity during walking, even for a few meters, associated with an inconstant burning sensation in correspondence to the surgical scar. The MRI performed 6 months after the digital amputation showed a flattening of the plantar arch with signs of overload at the fourth metatarsal bone head (Figure 4). The patient reports having changed his lifestyle with limitations in activities related to his free time such as running.

## 3. Discussion

MS is a common neuralgic pain affecting the forefoot and, in many cases, it leads to such debilitating pain that it modifies the person’s quality of life with regard to limitations during walking [42].

The management of MS is conventionally based on the initial use of non-operative treatments, and only in the case of their failure is surgical excision indicated. The treatment traditionally starts in a conservative manner with the use of orthotic devices, metatarsal pads, and analgesics. Ultrasound can confirm the diagnosis and offer immediate pain relief when combined with percutaneous injections. For patients not responding to initial conservative measures, the injection of corticosteroids with the addition of anesthetic, chemical neurolysis with alcohol, or radiofrequency ablation, can be performed as a non-surgical mini-invasive measure. However, as reported in Table 1, non-surgical procedures are not lacking in complications [33,36,37,38,39,43,44,45,46,47,48,49,50,51].

Although USGAI is one of the most popular non-surgical treatments, it also has a considerable number of complications, mostly minor and already well described in the literature [33,36,37,38,39,43,44,45].

In 2004, Fanucci et al. [33], injecting 0.5 mL of a 30% ethylic alcohol solution in the MN of a consecutive series of 40 patients, reported only minor complications: transitory and moderate pain at the site of injection, and a transitory plantar pain due to leakage of fluid. In 2007, Hughes et al. [36], using 0.5 mL of a 20% ethylic alcohol solution, described transitory plantar pain in 17 of 101 patients treated. In the same year, Mozena and Clifford [44], using 0.5 mL of a 4% dehydrated alcohol solution, recorded only minor complications: pain and erythema around the injection area. In 2012, Musson et al. [39] noted that 1 of 75 patients treated with 0.5 mL of a 20% ethylic alcohol solution developed symptoms of facial swelling and vomiting soon after the first treatment, consistent with an allergic reaction. In 2013, Gurdezi et al. [52] described severe pain and bruising at the injection site and numbness of the toes in their cohort. More recently, Samaila et al. [45], in a cohort of 115 patients treated by injections of 3 mL of a 6% phenol solution, reported a singular case of osteonecrosis of the third metatarsal head 2 years after the last alcoholization in addition to minor complications. Other studies documented similar complications: intolerable transient pain [34], mean local inflammatory reactions, short-lived pain at the moment of injection [35,37], skin depigmentation, plantar fat pad atrophy [47], perilesional scar [49], local cellulitis and hematoma [48,50,51]. Finally, Ortu et al. [38] reported that 3 of 200 patients treated by injection of 1 mL of a 47.5% alcohol solution developed skin necrosis with an involvement of subcutaneous tissue and peritendinous exposition, a known effect of alcohol therapy but not previously reported for ultrasound-guided alcohol injection for MN.

To the best of our knowledge, the complication described in this report is the first case reported in the modern scientific literature. For about twenty years, the use of USGAI has been proposed as an interesting and promising treatment option to surgery due to its tolerability, apparently low adverse events, and immediate walking after the procedure. Different reports about alcohol injections have demonstrated improvements in 69–90% of cases [33,39], and a 30–50% decrease in the size of the neuroma [33,36]. According to Lizano-Diez et al. [22]., the addition of a corticosteroid to the injection performed for the treatment of MS does not improve pain or function compared to a local anesthetic alone, whereas Matthews et al. found evidence of limited pain reduction following corticosteroid injections. According to a recent meta-analysis, the treatment of MS by corticosteroid injections is less effective than US-guided chemical neurolysis in terms of permanent pain relief and patient satisfaction. The USGAI is also repeatable: for some authors, a minimum of four injections is necessary to totally alleviate symptoms and reduce the risk of recurrence [36]. At present, there is not enough evidence to support the effectiveness of alcoholization for intermetatarsal neuroma, even if ultrasonography-guided, as the literature only reports a few small studies evaluating the effectiveness of the treatment [35].

Furthermore, the longest follow-up study available showed that alcohol injection did not offer a permanent resolution of symptoms for most patients: approximately one third of patients undergoing surgery, one third with pain recurrence, and only one third remaining pain free at five years follow-up [52]. Even if the operative excision of the neuroma has higher costs and a considerable risk of post-operative complications in 23% of cases (such as wound infection, hypersensitive scars, or keloids) [53], it remains the gold standard of treatment performed in patients who do not respond to conservative measures [1,31,33]. Santos reports that the highest post-alcohol injection adverse events occurred at alcohol concentrations of 30% and 50% [54], the same concentration used during the procedure described in this case report, where a rare complication of digital ischemia occurred after USGAI. However, there is no mention of correlation between the severity of complications and the concentration of alcohol injected in the literature (4–50%) [38]. It was shown that alcohol injection around the nerve produces chemical neurolysis by dehydration, necrosis, denaturation of proteins, and precipitation of protoplasm, inducing Wallerian degeneration and inhibiting neurotransmission [31,33,55]. However, the histological findings in surgical specimens of non-responder patients after alcohol sclerosing injection showed intra and perineural fibrosis, reduced cellularity, and sclerosis. These histological aspects demonstrate only partial damage of the nervous tissue caused by alcohol infiltration [35,36,37]. Further, Mazoch et al. [56], did not find any significant histological changes in rat sciatic nerve after injections of solutions of 4%, 20%, and 30% alcohol, raising questions about the efficacy of alcohol injections. Reflecting on the absence of literature on digital ischemia after USGAI, we speculated on why it occurred in this case. Probably, although the procedure was performed in the standardized way [35,37], the vascular axis was poorly recognized under ultrasound guidance and the alcoholization liquid was injected into it, causing a vascular thrombosis with consequent ischemia of the toe. Another hypothesis on the genesis of ischemia could be that alcohol injected into the vascular bed caused a massive lesion of the endothelium with consequent hemorrhage and inflammation and subsequent downstream ischemia. Finally, as suggested by some authors [38], the tissue necrosis at the distal level of the toe may have been caused by an excessive intra and perilesional resistance occurring during the injection, with subsequent extravasation of solution into the surrounding tissues until it progressively reached the distal part of the fourth toe, where the damage was irreversible.

According to this report, digital ischemia is a complication to consider in choosing the most appropriate treatment, although this represents the only case described. However, when compared to complications of other therapies, it is undoubtedly one of the worst sequalae. Digital amputation inevitably leads to a permanent change in the plantar arch, which is associated with constant metatarsalgia, progressive varism deformity of the fifth toe, and limitations during walking, with a consequent deterioration in the quality of life, unlike the complications already reported in the literature, which in most cases are temporary and easily treated [57].

## 4. Conclusions

This is the first report in the literature describing digital ischemia after USGAI for the treatment of MS. For this reason, from now on, this unusual complication should be included in the decision-making process when the clinician selects the most suitable treatment among the several proposed for this syndrome. Second, patients should be properly informed by the clinician about this potential sequalae before undergoing alcoholization.

## Figures and Tables

**Table 1:** Main complications of the non-surgical procedures described in the modern literature for Morton’s syndrome treatment (-: no reported).

Non-Surgical Invasive Techniques	Authors (Publication Year and Type)	Number of Complications (/) In Each Patient Cohort	Local Complications	Systemic Complications
	Fanucci et al. (2004) [33] Case Series	6/40	Transitory plantar pain due to leakage of fluid	-
Alcohol Injection (USGAI)	Hughes et al. (2007) [36] Case Series	18/101 17/101 1/101	Plantar pain Forefoot marrow edema	-
	Mozena & Clifford (2007) [44] Case Series	3/42	Pain and Erythema around the injection area	-
	Musson et al. (2012) [39] Case Series	1/75	Pain Swelling	Allergic reaction
	Perini et al. (2016) [37] Case Series	178/220 2/220 176/220	Symptoms worsening Hypo/Anaesthesia of the innervated area	-
	Samaila et al. (2020) [45] Case Series	1/115	Osteonecrosis of the third metatarsal head	-
	Ortu et al. (2022) [38] Case Series	3/200	Skin necrosis	-
Capsaicin Injection	Campbell et al. (2016) [46] Case Series	30/30 4/30 3/30	Severe Post-Injection Pain	Nausea Headache
Corticosteroid Injection	Choi et al. (2021) [47] Review	10/294 3/51 2/216	Skin Depigmentation Skin Atrophy Plantar Fat Pad Atrophy	-
ESWT	Auersperg et al. (2020) [48] Review	-	Redness Superficial Hematoma	-
Laser Therapy	Gimber et al. (2017) [49] Case series	-	Perilesional Scar	-
Percutaneous Cryoablation	Cazzato et al. (2016) [50] Case Series	1/20	Local Cellulitis around cryo-probe entry point	-
RFA	Deniz et al. (2015) [51] Case Series	2/20	Superficial Cellulitis and moderate Hematoma	-

## Data Availability

The dataset supporting the conclusions of this review is available upon request to the corresponding author.
